# Supplementary material for: Mechanisms governing the pioneering and redistribution capabilities of the non-classical pioneer PU.1
Source: Nat Commun. 2020 Jan 21;11:402. doi: 10.1038/s41467-019-13960-2 (PMC6972792; doi:10.1038/s41467-019-13960-2)
Supplement: Supplementary file 7 — Source data [file 41467_2019_13960_MOESM7_ESM.zip › Source_Data/FigureS5/FigureS5B_MotifsETS1specificPeaks/homerResults/motif5.similar.html]

motif5

## Information for motif5

T
A
G
C
A
T
G
C
G
C
T
A
C
T
A
G
T
A
C
G
C
G
T
A
A
C
G
T
A
C
T
G
  
Reverse Opposite:  

G
T
A
C
T
G
C
A
A
C
G
T
A
T
G
C
A
G
T
C
C
G
A
T
A
T
C
G
A
T
C
G
  

|  |  |
| --- | --- |
| p-value: | 1e-89 |
| log p-value: | -2.062e+02 |
| Information Content per bp: | 1.818 |
| Number of Target Sequences with motif | 358.0 |
| Percentage of Target Sequences with motif | 31.54% |
| Number of Background Sequences with motif | 687.1 |
| Percentage of Background Sequences with motif | 8.42% |
| Average Position of motif in Targets | 103.5 +/- 47.7bp |
| Average Position of motif in Background | 103.9 +/- 57.4bp |
| Strand Bias (log2 ratio + to - strand density) | 0.1 |
| Multiplicity (# of sites on avg that occur together) | 1.14 |
| Motif File: | file (matrix) reverse opposite |

### Similar de novo motifs found

|  |  |  |  |  |  |  |  |
| --- | --- | --- | --- | --- | --- | --- | --- |
| Rank | Match Score | Redundant Motif | P-value | log P-value | % of Targets | % of Background | Motif file |
| 1 | 0.921 | A G T C A G T C A T G C G C T A C A T G C T A G C T G A C A G T C A T G | 1e-52 | -120.776677 | 38.06% | 17.28% | motif file (matrix) |
| 2 | 0.793 | C A T G C T A G G T A C G T C A A G C T A C T G A G T C A G C T A C T G A C T G | 1e-45 | -105.153198 | 21.59% | 7.05% | motif file (matrix) |
| 3 | 0.786 | C G T A C T G A A G C T G T A C G T A C A G T C C T G A A C T G T A C G C T G A A C G T C A T G G A T C | 1e-45 | -104.257491 | 6.87% | 0.42% | motif file (matrix) |
| 4 | 0.848 | C T A G A G T C C T G A A C G T A G T C A G T C A G C T A C T G G T A C | 1e-43 | -100.169822 | 11.01% | 1.84% | motif file (matrix) |
| 5 | 0.863 | C T A G G T A C G T C A A G C T A C T G A G T C A G C T A C T G T C A G | 1e-42 | -97.276854 | 20.79% | 6.97% | motif file (matrix) |
| 6 | 0.839 | A C T G A T G C A T G C A G T C C G T A A C T G A T G C G C T A A C G T A C T G G A T C | 1e-20 | -47.219171 | 4.58% | 0.64% | motif file (matrix) |
| 7 | 0.801 | A T G C G T A C T C G A G T C A C G T A A G T C C G T A A C G T A G T C A G T C A C G T A C T G A G C T A C T G | 1e-14 | -34.336908 | 1.94% | 0.08% | motif file (matrix) |
